# Comparing the efficacy and safety of medications in adults with hypertrophic cardiomyopathy: a systematic review and network meta-analysis

**DOI:** 10.3389/fcvm.2023.1190181

**Published:** 2023-08-14

**Authors:** Keying Mi, Sijia Wu, Chanyuan Lv, Yongkang Meng, Wenchao Yin, Hongkai Li, Jiangbing Li, Haitao Yuan

**Affiliations:** ^1^ Department of Cardiology, Shandong Provincial Hospital, Shandong University, Jinan, China; ^2^ JiNan Key Laboratory of Cardiovascular Disease, Jinan, China; ^3^ Department of Biostatistics, School of Public Health, Cheeloo College of Medicine, Shandong University, Jinan, China; ^4^ Institute for Medical Dataology, Cheeloo College of Medicine, Shandong University, Jinan, China; ^5^ Department of Cardiology, Shandong Provincial Hospital Affiliated to Shandong First Medical University, Jinan, China

**Keywords:** hypertrophic cardiomyopathy, system review and meta-analysis, left ventricular outflow tract gradient, medications, frequentist network meta-analysis

## Abstract

**Background:**

Hypertrophic cardiomyopathy (HCM) is the most common genetic heart disease. The purpose of this study was to evaluate the efficacy and safety of several medications and recommend better drug treatments for adults with HCM.

**Methods:**

A review of PubMed, Embase, the Cochrane Controlled Register of Trials (CENTRAL), ClinicalTrials.gov and CNKI databases was conducted for studies on the efficacy and safety of drugs for adults with HCM. A frequentist random effects model was used in this network analysis.

**Results:**

This network meta-analysis included 7 studies assessing seven medications, 6 studies evaluating monotherapy and 1 study evaluating combination therapy. Based on the network meta-analysis results, xiaoxinbi formula plus metoprolol (MD −56.50% [−72.43%, −40.57%]), metoprolol (MD −47.00% [−59.07%, −34.93%]) and mavacamten (MD −34.50% [−44.75%, −24.25%]) significantly reduced the resting left ventricular outflow tract gradient (LVOTG) in comparison with placebo. Resting LVOTG could also be reduced with N-acetylcysteine (NAC). The incidence of adverse drug reactions was not significantly different between the placebo group and the treatment group.

**Conclusion:**

For adults with HCM, the top 4 treatments included xiaoxinbi formula plus metoprolol, metoprolol, mavacamten and NAC.

**Systematic Review Registration**: [https://www.crd.york.ac.uk/prospero/display_record.php?RecordID=374222], identifier [CRD42022374222].

## 1. Background

Hypertrophic cardiomyopathy (HCM) is a genetic disease which affects cardiac myocytes. HCM is characterized by cardiac hypertrophy unrelated to loading conditions, with a nondilated left ventricle and a normal or elevated ejection fraction (1). Over 1,400 mutations have been found in eleven genes that encode cardiac sarcomere proteins (2). Numerous previous studies have shown that approximately one in 500 adult subjects worldwide suffers from HCM (3, 4). Men are more likely to be affected than women, with a prevalence of approximately 60%. Even so, the mortality in females is higher than that in males (5). Importantly, HCM can result in sudden arrhythmic death, heart failure, and atrial fibrillation (AF) (6).

HCM can be treated with pharmacologic therapy, lifestyle intervention, surgical treatment and gene therapy. Currently, drug treatment mainly consists of beta-blockers, non-dihydropyridine calcium receptor antagonists, cardiac myosin inhibitors, ion channel inhibitors, angiotensin II receptor blockers and disopyramide (7). However, all of these drug treatments have side effects. As one of the most commonly used and effective drugs, non-vasodilating beta-blockers effectively reduce left ventricular outflow tract (LVOT) obstruction (LVOTO) and prolong diastole (8). If a patient has an intolerance or a contraindication to beta-blockers, then non-dihydropyridine calcium channel blockers may be a good alternative. As recommended by the guidelines, beta-blockers, non-dihydropyridine calcium channel blockers and disopyramide are considered drugs that reduce LVOTO and improve overall heart function (9). Through ion exchange, HCM produces enhanced late sodium current (I_Na_L) activity owing to enzyme-mediated sodium-channel phosphorylation, which increases intracellular sodium (Na^+^) as well as calcium (Ca^2+^) overload (10). There may be an underlying mechanism for abnormal muscle contractions and diastolic dysfunction caused by prolonged intracellular Ca^2+^ transients and higher diastolic [Ca^2+^]_i_. And increased arrhythmogenicity can be attributed to longer action potentials and greater incidences of early afterdepolarizations (EADs) and delayed afterdepolarizations (DADs) (11). In theory, ion channel inhibitors, including ranolazine and eleclazine could counteract diastolic dysfunction, stimulation of microvascular function, and myocardial relaxation by inhibiting late sodium current activity (10). Studies on sarcomeric HCM mice have shown that hypertrophy and fibrosis are largely mediated by transforming growth factor-beta (TGF-b) (12). Angiotensin II receptor blockers (ARBs) are known to inhibit TGF-b activation and may slow the progression of HCM or even prevent its occurrence, according to a recent clinical trial (13). Recently, a new specific therapy, cardiac myosin inhibitors, has emerged for the treatment of HCM. Mavacamten (MYK-461) reduces cardiac contractility by reversible, selective inhibition of myosin ATPase (14). In the present study, mavacamten resulted in a greater increase in peak oxygen consumption (pVO_2_) (+1.4 ml/kg per min, *p* = 0.0006), greater reductions in post-exercise left ventricular outflow tract gradient (LVOTG) (−36 mmHg, *p* < 0.0001), and similar safety results compared to placebo (15). Surgical intervention may be required for patients who retain symptoms following guideline-guided management and treatment, including myectomy, septal ablation and cardiac resynchronization therapy. As these procedures are invasive, they need technical experience to be performed adequately (9, 16).

Since surgery is a complicated procedure, patients are more likely to be treated with medications to alleviate their symptoms than with surgery. Considering the increasing number of adults who have HCM, more effective medications should be given to reduce LVOTG, with fewer adverse reactions and complications. Thus, the aim of this systematic review and network meta-analysis was to provide an overview of the most commonly prescribed medications for the treatment of adults with HCM and provide suggestions for additional pharmaceutical management by assessing the safety and efficacy of several medications.

## 2. Material and methods

### 2.1. Search strategy

Our protocol was registered in PROSPERO (CRD42022374222), and our processes and outcomes were reported according to the PRISMA (Preferred Reporting Items for Systematic Reviews and Meta-analyses extension statement for network meta-analyses) guidelines. A comprehensive search of the PubMed, Embase, Cochrane Library, ClinicalTrials.gov and CNKI databases was performed without language limitations from the date of inception until November 2022. After searching all randomized clinical trial (RCT) studies under the title of hypertrophic cardiomyopathy, studies were selected based on inclusion and exclusion criteria.

### 2.2. Selection criteria and eligibility criteria

The inclusion criteria were as follows: (1) Research type: RCTs were analyzed; (2) Population: studies enrolling participants with HCM aged 18 years or older were included; (3) Intervention: studies that compared at least two different medications (including placebo) were considered. Intervention could be a single medication or a combination of medications; (4) Comparisons: a placebo or other therapeutic agent. Direct and indirect comparisons formed a network between drugs; (5) Outcomes: studies were included if they reported changes in resting LVOTG compared with control groups after intervention. As part of the safety evaluation, the incidence of drug-related adverse events was also assessed.

Studies were independently searched and screened by two researchers (YM and WY). A discussion with another reviewer (HL) was held to resolve any disagreements.

### 2.3. Data extraction

For each of the included studies, the following variables were independently extracted by two investigators (Keying Mi and Sijia Wu): research characteristics (published year, country, duration of medication), research type (randomized controlled trials), participant characteristics (sample size, HCM diagnostic criteria, demographics), and outcomes (change in resting LVOTG from baseline, incidence of adverse events). For any disagreements, the reviewers discussed or consulted with a third reviewer (Jiangbing Li).

### 2.4. Risk of bias assessment

Using the risk of bias assessment tool from the Cochrane Collaboration, two researchers (Keying Mi and Sijia Wu) independently evaluated the included studies' risk of bias, taking into account factors such as random sequence generation, allocation concealment, blinding, missing outcome data and selective reporting of outcomes (17). Each domain was assigned a risk of bias rating of low, unclear, or high. A third reviewer (Jiangbing Li) resolved any disagreements.

### 2.5. Data synthesis

Using a frequentist network meta-analysis, the MD and 95% confidence intervals (CIs) were calculated for changes in resting LVOTG levels and the odds ratio (OR) and its 95% CIs for dichotomous outcomes. Cochran's *Q* statistic was examined as a method to quantify heterogeneity. The model to be used was determined based on Cochran's *Q* statistic. Due to the consistency between the fixed effects model and random effects model results, this analysis was conducted with a random effects model. In this study, our network consistency was evaluated both locally by comparing direct and indirect evidence, as well as globally by using the model of design-by-treatment interaction (18, 19). *P*-scores were based solely on network meta-analysis point estimates and standard errors were used to rank treatments. An average of all competitive treatments was used to determine whether one treatment was superior to another (20).

A subgroup analysis was also planned. Obstructive hypertrophic cardiomyopathy and non-obstructive hypertrophic cardiomyopathy are two types of HCM. For the subgroup analysis, all treatments were divided into an obstructive hypertrophic cardiomyopathy group and a non-obstructive HCM group, with the results compared to the previous results to determine consistency.

## 3. Results

### 3.1. Baseline characteristics

**Figure 1:**
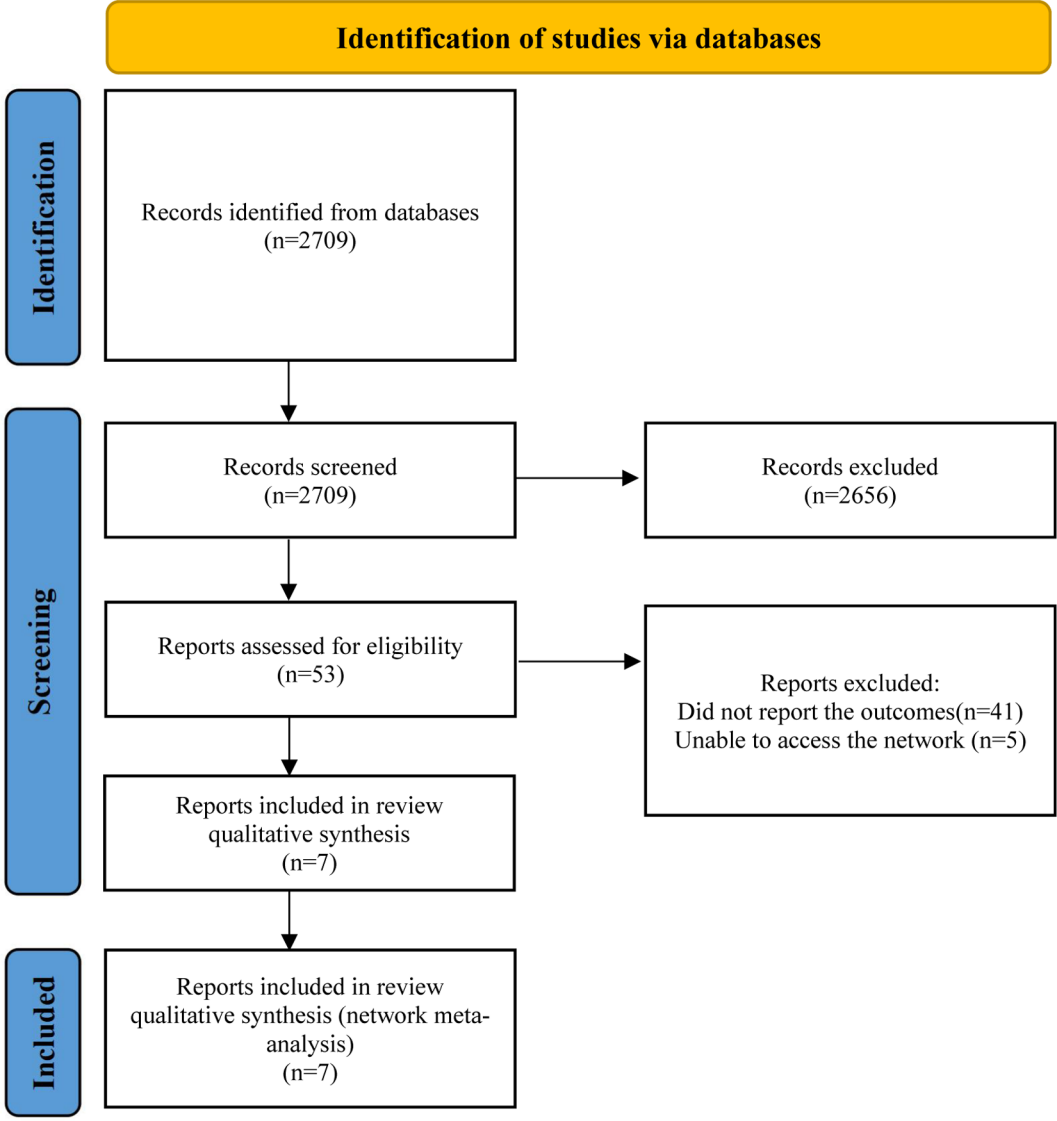
Flow diagram.

This systematic review and network meta-analysis included seven studies (377 patients), consisting of two published papers (21, 22) and five clinical trials (NCT04349072, NCT01696370, NCT00430833, NCT03532802, NCT01537926). A detailed description of the selection process is presented in Figure 1. Table 1 summarizes the characteristics of the included studies. There was less than a 12-month study period in all studies. Among the studies, five were double-blinded, one was quadruple-blinded and one may be open-label. In total, these studies examined seven drugs or drug combinations for the treatment of HCM, including mavacamten, trimetazidine, candesartan, metoprolol, N-acetylcysteine (NAC), verapamil, xiaoxinbi formula plus metoprolol.

**Table 1:** The characteristics of included studies.

Study	Blinding setting	Study period (weeks)	Interventions	Sample	Baseline resting LVOTG (mmHg)	HCM mutation types
NCT04349072	Double blind	16	Mavacamten	56	51.2 ± 31.4	Not mentioned
			Placebo	56	46.3 ± 30.5	
NCT01696370	Double blind	12	Trimetazidine	26	6.5 ± 4.2	Not mentioned
			Placebo	23	8.2 ± 15.9	
NCT00430833	Double blind	48	Candesartan	12	7.5 ± 3.1	β-MHC gene mutations, cMYBPC gene mutations, TnI gene mutations and myofilament genotype-negative
			Placebo	12	9.2 ± 6.3	
NCT03532802	Double blind	2	Metoprolol	29	21–97	Not mentioned
			Placebo	29	21–97	
NCT01537926	Quadruple	48	NAC	29	13.12 ± 23.48	ACTC1 gene mutations, ACTN2 gene mutations, MYBPC3 gene mutations, MYH7 gene mutations, MYL2 gene mutations, TNNT2 gene mutations, TTN gene mutations
			Placebo	13	8.85 ± 21.76	
Gistri et al., 1994 (22)	Double blind	8.0 ± 2.4	Verapamil	10	15 ± 14	Not mentioned
			Placebo	10	19 ± 19	
Zhang et al., 2017 (21)	Open label^a^	13	Xiaoxinbi formula + metoprolol	37	45.3 ± 21.6	Not mentioned
			Metoprolol	35	41.5 ± 18.7	

Except where indicated, data are presented as mean ± SD or minimum-maximum. NAC, N-acetylcysteine; HCM, hypertrophic cardiomyopathy.

^a^ Not mentioned in the article, derived from the method.

### 3.2. Risk of bias assessment

Supplementary Figure S1 features an assessment of the risk of bias in the included studies. The biases of all included studies [(21, 22), NCT04349072, NCT01696370, NCT00430833, NCT03532802] were assessed as low risk.

### 3.3. Network meta-analysis

**Figure 2:**
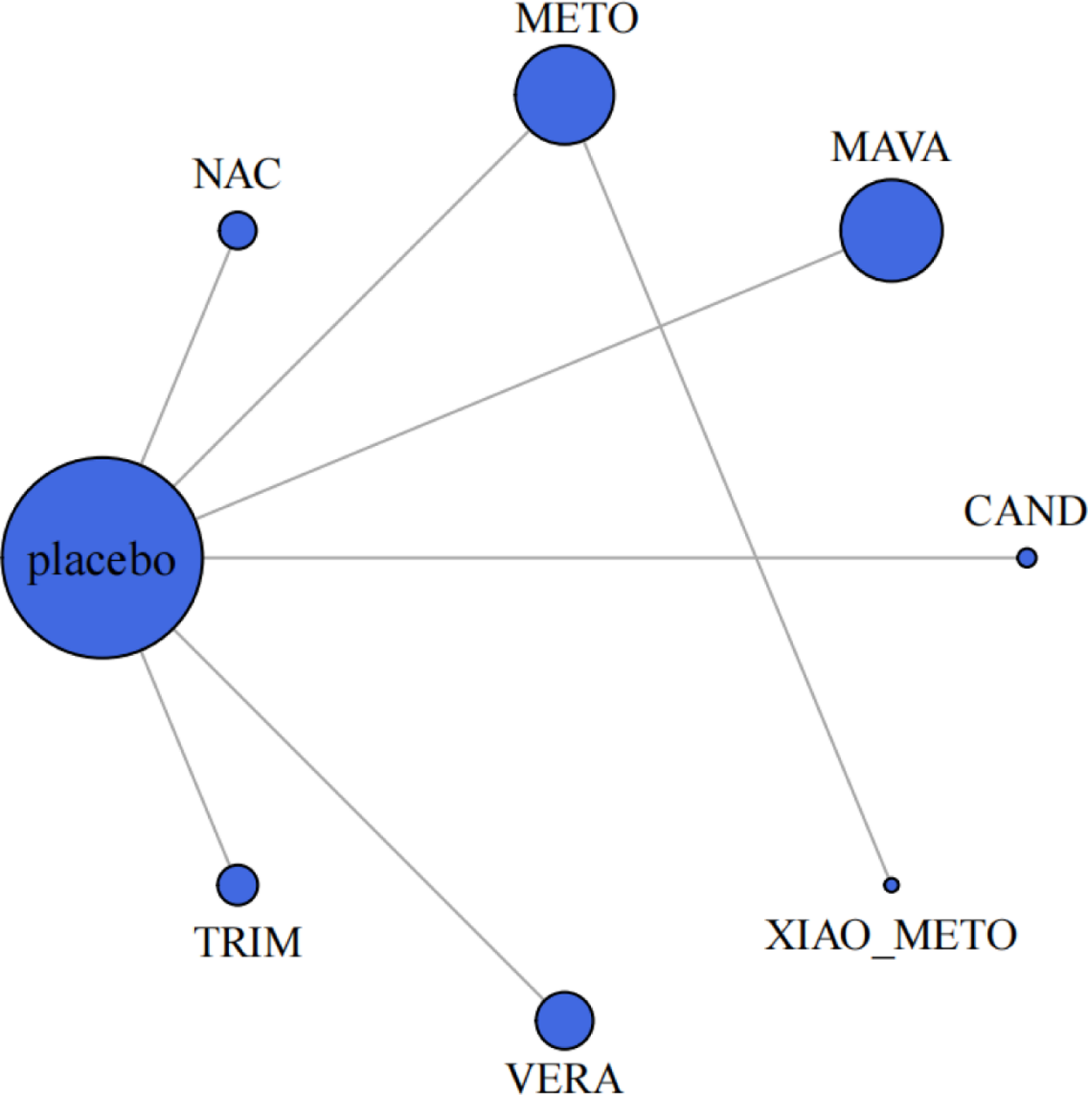
Meta-analysis networks for change in resting LVOTG level. Each circle indicates a treatment node. Lines connecting 2 nodes represent direct comparisons between 2 treatments. The size of the nodes is proportional to the number of trials evaluating each treatment. The thickness of the lines is proportional to the number of trials directly comparing the 2 connected treatments. NAC, N-Acetylcysteine; METO, metoprolol; MAVA, mavacamten; CAND, candesartan; XIAO_METO, xiaoxinbi formula + metoprolol; VERA, verapamil; TRIM, trimetazidine.

In our network meta-analysis, medications (mavacamten, trimetazidine, candesartan, metoprolol, NAC, verapamil, xiaoxinbi formula) and their combinations were evaluated (Figure 2). A random effects model was applied in this meta-analysis.

#### 3.3.1. Primary outcomes

**Figure 3:**
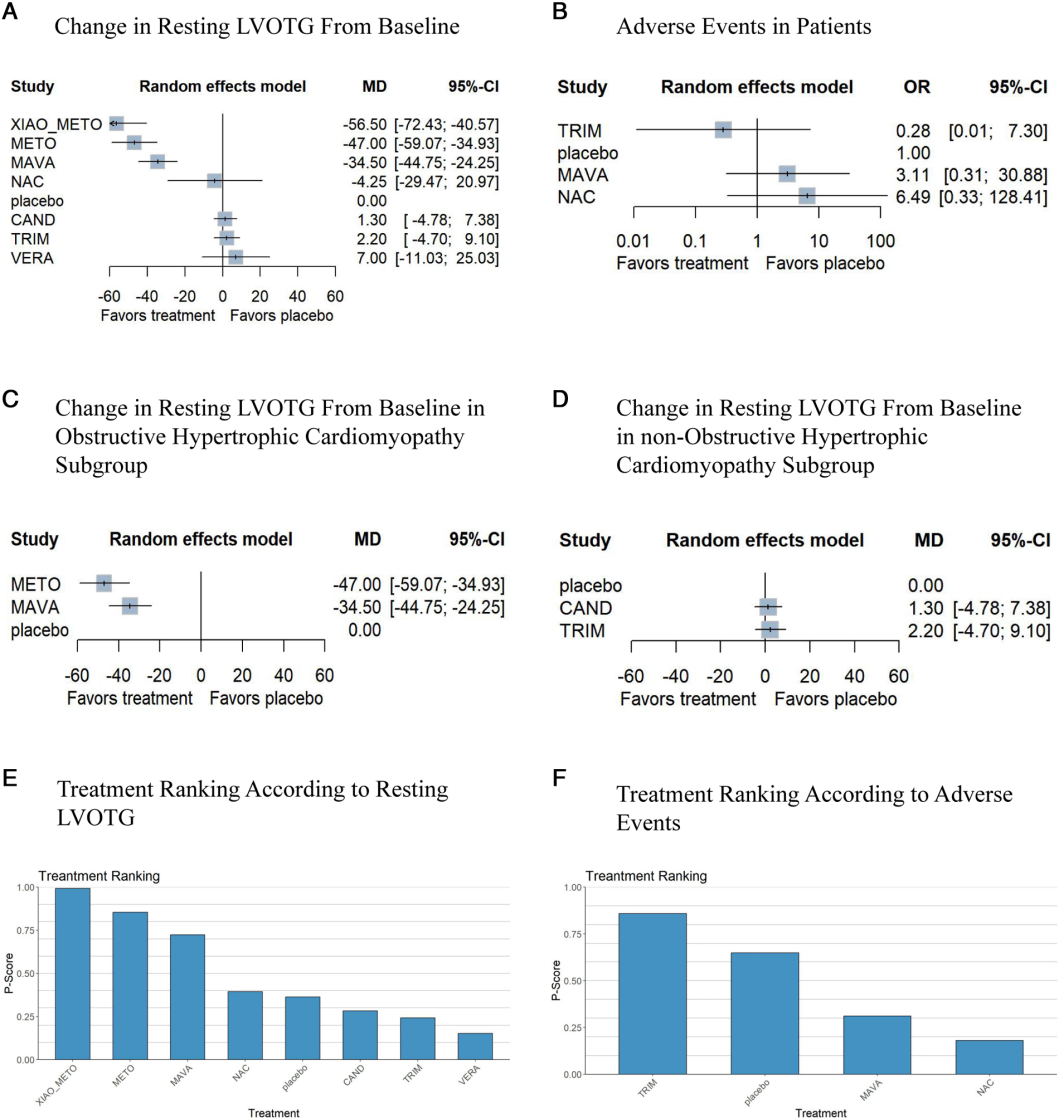
Network meta-analysis results for the primary outcomes compared with placebo. Treatments are presented according to their effect estimate compared with a placebo. Effects sizes are presented as MD or OR with 95% CIs. MD, mean difference; OR, odds ratio; NAC, N-Acetylcysteine; METO, metoprolol; MAVA, mavacamten; CAND, candesartan; XIAO_METO, xiaoxinbi formula + metoprolol; VERA, verapamil; TRIM, trimetazidine. (**A**) the change in resting LVOTG from the baseline of the random effects model; (**B**) adverse events of patients of the random effects model; (**C**) the change in resting LVOTG from the baseline of the random effects model in obstructive hypertrophic cardiomyopathy subgroup; (**D**) the change in resting LVOTG from baseline of random effects model in non-obstructive hypertrophic cardiomyopathy subgroup; (**E**) treatment ranking according to resting LVOTG of random effects model; (**F**) treatment ranking according to adverse events of random effects model.

A total of 377 patients from seven studies were studied for changes in resting LVOTG from baseline. Based on a random effects model, compared with placebo, the diminution of resting LVOTG was obviously larger in xiaoxinbi formula + metoprolol (MD −56.50% [−72.43%, −40.57%]), metoprolol (MD −47.00% [−59.07%, −34.93%]), mavacamten (MD −34.50% [−44.75%, −24.25%]), NAC (MD −4.25% [−29.47%, 20.97%]), respectively (Figure 3A). Compared to placebo, in this model, three drugs did not show significant differences in resting LVOTG changes from baseline, including candesartan (MD 1.30% [−4.78%, 7.38%]), trimetazidine (MD 2.20% [−4.70%, 9.10%]) and verapamil (MD 7.00% [−11.03%, 25.03%]).

In addition, xiaoxinbi formula + metoprolol was the most promising intervention for decreasing resting LVOTG, followed by metoprolol, and mavacamten (Figure 3E).

Based on the available three studies, three drugs were compared in terms of adverse events (Figure 3B). In comparison with placebo, trimetazidine, mavacamten and NAC showed no significant differences in the occurrence of adverse events (Figure 3F).

#### 3.3.2. Secondary outcomes

##### 3.3.2.1. Efficacy outcomes

The resting LVOTG of patients was reported to have changed from baseline in the seven studies. A significant reduction in resting LVOTG was observed using xiaoxinbi formula + metoprolol, metoprolol, mavacamten, and NAC in the random effects model. In contrast, trimetazidine, candesartan and verpamil yielded different results in this model. Additionally, xiaoxinbi formula + metoprolol was found to be the most effective drug treatment for decreasing resting LVOTG, while metoprolol was the second most effective intervention (Figure 3A).

##### 3.3.2.2. Safety outcomes

Three different medications were compared to a placebo to determine their safety, as only three studies demonstrated the occurrence of adverse events. In this network meta-analysis, trimetazidine, mavacamten and NAC showed no significant difference from placebo (Figure 3B). As shown in Figure 3F, treatments were ranked based on their *p*-score for safety outcomes.

### 3.4. Subgroup analyses

In the subgroup analysis, to examine the efficacy of the different medications, the patients were divided into two groups: those with obstructive hypertrophic cardiomyopathy and those with non-obstructive hypertrophic cardiomyopathy. In the obstructive hypertrophic cardiomyopathy subgroup, metoprolol showed the best efficacy, followed by mavacamten (Figure 3C). However, trimetazidine and candesartan did not demonstrate a therapeutic benefit compared to placebo in the non-obstructive hypertrophic cardiomyopathy group (Figure 3D).

## 4. Discussion

Beta-blockers and non-dihydropyridine calcium channel blockers are the first recommended treatments for HCM. Recently, the FDA approved mavacamten for the targeted treatment of HCM. However, studies comparing the efficacy and safety of different drug therapies in adults with HCM are rare.

In this study, a random effects model was used to evaluate the effects of seven drugs or drug combinations on changes in resting LVOTG and the incidence of adverse events among patients with HCM. AF, palpitations, dizziness, and headache are some of the most common adverse effects of drugs used to treat HCM. In this study, three drugs for which adverse events had been reported were compared for their safety. Based on our network meta-analysis of the seven studies, significant reductions were found in resting LVOTG for xiaoxinbi formula + metoprolol, metoprolol, mavacamten and NAC. According to the results of the *p*-score ranking, xiaoxinbi formula plus metoprolol, metoprolol, mavacamten and NAC remained the top four treatments. Furthermore, no significant differences were observed between the comparison group and the placebo group with respect to adverse events. While xiaoxinbi formula + metoprolol and metoprolol had superior efficacy compared to other treatments in our study, their safety requires additional validation since adverse events were not compared in our study. In a subgroup analysis, metoprolol demonstrated the best efficacy in patients with obstructive hypertrophic cardiomyopathy. Additionally, there was no significant difference in efficacy between the drugs (candesartan and trimetazidine) and placebo in the non-obstructive hypertrophic cardiomyopathy subgroup.

Metoprolol treatment for HCM has been demonstrated to be safe and effective (23). In the 2020 AHA/ACC guidelines, metoprolol was recommended as a class 1 medication (9). Nonetheless, few studies have examined the occurrence of adverse events during metoprolol treatment. Through their negative inotropic and chronotropic effects, beta blockers reduce tachycardia and ventricular contractility, increasing passive ventricular filling and diastolic filling time (24). Our study indicated that metoprolol had the second-highest efficacy, which is consistent with the recommendations in the guidelines. In obstructive hypertrophic cardiomyopathy, an elevated LVOTG is often accompanied by high myocardial contractility and strong adrenergic stimulation (25). Additionally, due to microvascular ischaemia, diastolic dysfunction, and elevated filling pressures, non-obstructive hypertrophic cardiomyopathy patients experience angina and dyspnoea (9). Through the above pharmacological mechanisms, beta blockers are capable of reducing LVOTG and relieving symptoms. In traditional Chinese medicine, xiaoxinbi formula has been shown to significantly improve cardiac function in combination with Western medicine. Based on direct and indirect comparisons, xiaoxinbi formula plus metoprolol was most effective in treating HCM. Nevertheless, the mechanism of xiaoxinbi formula treatment for HCM is unclear and may be related to all-cause multitargeted therapy in traditional Chinese medicine, the mechanism of which needs further study (21). Apart from these medications, NAC was also effective in treating HCM compared with placebo in our study. Researchers found that NAC reversed myocardial hypertrophy and fibrosis in an animal model of HCM, and improved diastolic function indices. Marian AJ et al. found that NAC reduced left ventricular mass and LVOTG (26). The reduction in myocardial hypertrophy may reduce the degree of LVOTO, and LVOTG will be reduced as well. It might act by reducing oxidative stress, which is increased in patients with HCM (26). Conversely, metoprolol's target may play a greater role in disease development, which may explain its superiority over NAC. HCM can also be treated with mavacamten, a recently developed targeted therapy. The PIONEER-HCM (Pilot Study Evaluating MYK-461 in Subjects with Symptomatic Hypertrophic Cardiomyopathy and Left Ventricular Outflow Tract Obstruction; NCT02842242) study, a phase 2, open-label study, showed significant reductions in post-exercise LVOT gradients in obstructive hypertrophic cardiomyopathy patients treated with mavacamten (27). A meta-analysis revealed that compared to placebo, mavacamten was associated with improved NYHA functional class and pVO_2_ (28). It reduces hypercontractility and improves myocardial energetics by reversibly inhibiting actin-myosin cross-bridging (29). There are three different functional states of myosin: the active cycling state, which involves actin-activated ATP hydrolysis with a rapid ATP turnover rate of a mere one second; the disordered relaxed (DRX) state, is the ATP turnover state of myosin without actin, in which ATP turnover times are extremely slow at 30s; the third state, the super-relaxed (SRX) state, has even longer ATP turnover times (30). Under resting muscle conditions, DRX state and SRX state are in dynamic equilibrium. The drug mavacamten significantly reduced DRX, enhancing SRX (31). Mavacamten treats HCM by altering the DRX↔SRX state equilibrium and influencing muscle contractility and energetics (30). In contrast to beta blockers, which improve symptoms but do not treat HCM specifically, mavacamten is emerging as a targeted treatment for this disease. Study results revealed that metoprolol was more effective than mavacamten since the LVOTG reduction was greater with metoprolol (−47.00% vs. −34.50%), and the potential mechanism may be that the two drugs regulate myocardial contractility to varying degrees. Metoprolol reduces heart rate and myocardial contractility by blocking adrenergic receptor, and mavacamten regulates myocardial contractility by increasing SRX and improving unbalanced DRX:SRX ratio. Future clinical studies directly comparing the two drugs may be required to further validate our results. And deeper basic research is needed to compare the extent to which the action mechanisms of the two drugs play a role in the occurrence and development of the disease, providing further evidence for better diagnosis and treatment of HCM in the future.

For other drugs, the drug candesartan is classified as an angiotensin II receptor (AT1) blocker. Both cardiomyocytes and fibroblasts express AT1 receptors on their membranes, which mediate hypertrophic and fibrotic responses in the cardiac chamber (4). The treatment may affect the hypertrophy of the left ventricle, thereby changing the degree of obstruction of the left ventricle outflow tract. In HCM patients, left ventricular fibrosis is an important pathological feature. However, it remains unclear what mechanisms lead to cardiac fibrosis when HCM mutations occur (4). This may account for the lack of a significant difference in the efficacy of candesartan compared to placebo. As a result, more basic research and cohort studies are needed to verify the efficacy and mechanism of candesartan in the treatment of HCM. Trimetazidine, an inhibitor of free fatty acid oxidation, causes glucose to be utilized by cardiac and muscle tissues to regulate myocardial metabolism (32). Cardiac energetics are abnormal in patients with HCM. Research shows that impaired inorganic phosphate metabolism is observed in patients carrying HCM-related sarcomeric mutations (33). This target is not responsive to trimetazidine, and therefore, the efficacy of trimetazidine is not significantly different from that of the placebo. Verapamil is a non-dihydropyridine calcium channel blocker. In cardiac myocytes, it inhibits L-type calcium channels, exerting a negative inotropic and chronotropic effect (4). Contrary to the guideline recommendations, verapamil was not more effective than the placebo in this study because the LVOTG was increased when verapamil was administered compared to the placebo (7.00% vs. 0%). This may have been due to the inclusion of patients with milder symptoms and the use of smaller doses of verapamil.

To assess the effectiveness of treatment for different types of HCM, a subgroup analysis was conducted. In the subgroup analysis, not all of the drugs were included, but the efficacy rankings did not change. Among the adverse events analyzed in this study were AF, palpitations, dizziness, and headaches. Based on this analysis, there were no significant differences between the comparison group and the placebo group in terms of adverse events. Moreover, Torsades de pointes (TdP) is also an important adverse event, but it was not analyzed in this study due to data limitations. And it is associated with a prolonged QT interval in the ECG (34). In an RCT study, no nonsustained ventricular tachycardia was observed in the mavacamten treatment group, but no data about the incidence of TdP was reported (35). Trimetazidine has been shown to shorten QT intervals in research (36). As a result, TdP is unlikely to occur. Also, verapamil is the first-line treatment for sc-TdP (37). There are other HCM treatments that can prolong the QTc interval, such as disopyramide and amiodarone, but these two treatments were not included in this network study due to data limitations. As TdP can have serious consequences, future research should also focus on the probability of TdP occurrence in order to better measure drug safety.

Currently, HCM targeted treatments are increasingly mutation-specific. Toepfer et al. found as HCM can be caused by different gene mutations, the central pathological mechanism differs (38). Toepfer et al. showed that alternate calcium regulation is the central pathomechanism of thin filament variants TNNT2R92Q/+ and TNNI3R21C/+ (38). Sarkar et al. reported SRX is decreased by mutations R403Q and R663H in the HCM (39). Individual specific treatments will be possible due to the different pathogenesis of HCM caused by different mutations. The efficacy of this therapy needs to be evaluated by further research.

Despite this, there were some limitations in our study. First, in our analysis, only seven studies were included, which constituted a relatively small sample size. As a result of the limited number of studies, it is not possible to choose a more common Bayesian model to analyze the data. The Bayesian model analysis is recommended if there will be future larger sample studies or therapeutic research targeting specific gene mutations. Therefore, more studies are needed to confirm our findings. Furthermore, as part of our analysis, only changes in resting LVOTG—the most representative indicator—as an indicator of efficacy was analysed, whereas several clinical studies chose changes in pVO_2_ as an observed endpoint; thus, additional studies are required to confirm our conclusions. Moreover, according to this research, xiaoxinbi formula plus metoprolol was found to be the most effective treatment for HCM. Due to the small sample size and the uncertainty concerning the mechanism by which the xiaoxinbi formula treats HCM, more clinical and basic researches are necessary to verify its effectiveness. In addition, limited data made it difficult to compare different populations and age groups. Last, the occurrence of adverse events was not reported for all drugs in our study, limiting our ability to determine the safety of the medications.

## 5. Conclusions

Our results indicated that the four most effective medications for HCM in adults are xiaoxinbi formula + metoprolol, metoprolol, mavacamten and NAC. In different subgroups, drug efficacy ranked the same. The use of candesartan, trimetazidine and verapamil had no effect on reducing LVOTG. Therefore, they cannot delay the progression of the disease and provide no benefit over a placebo in treating HCM. There is a need for further research to assess the efficacy of metoprolol versus mavacamten. What's more, research on basic and molecular levels can be complementary or informative for future statistical studies. In this way, they can reveal how drugs work and develop more targeted and effective treatment methods for HCM at the molecular level.

## Data Availability

The original contributions presented in the study are included in the article/**Supplementary Material**, further inquiries can be directed to the corresponding authors.

## Supplementary material

Supplementary Figure S1 Summary of bias risk.
