# Supplementary figures and images for: Comparing the efficacy and safety of medications in adults with hypertrophic cardiomyopathy: a systematic review and network meta-analysis
Source: Front Cardiovasc Med. 2023 Aug 14;10:1190181. doi: 10.3389/fcvm.2023.1190181 (PMC10461399; doi:10.3389/fcvm.2023.1190181)

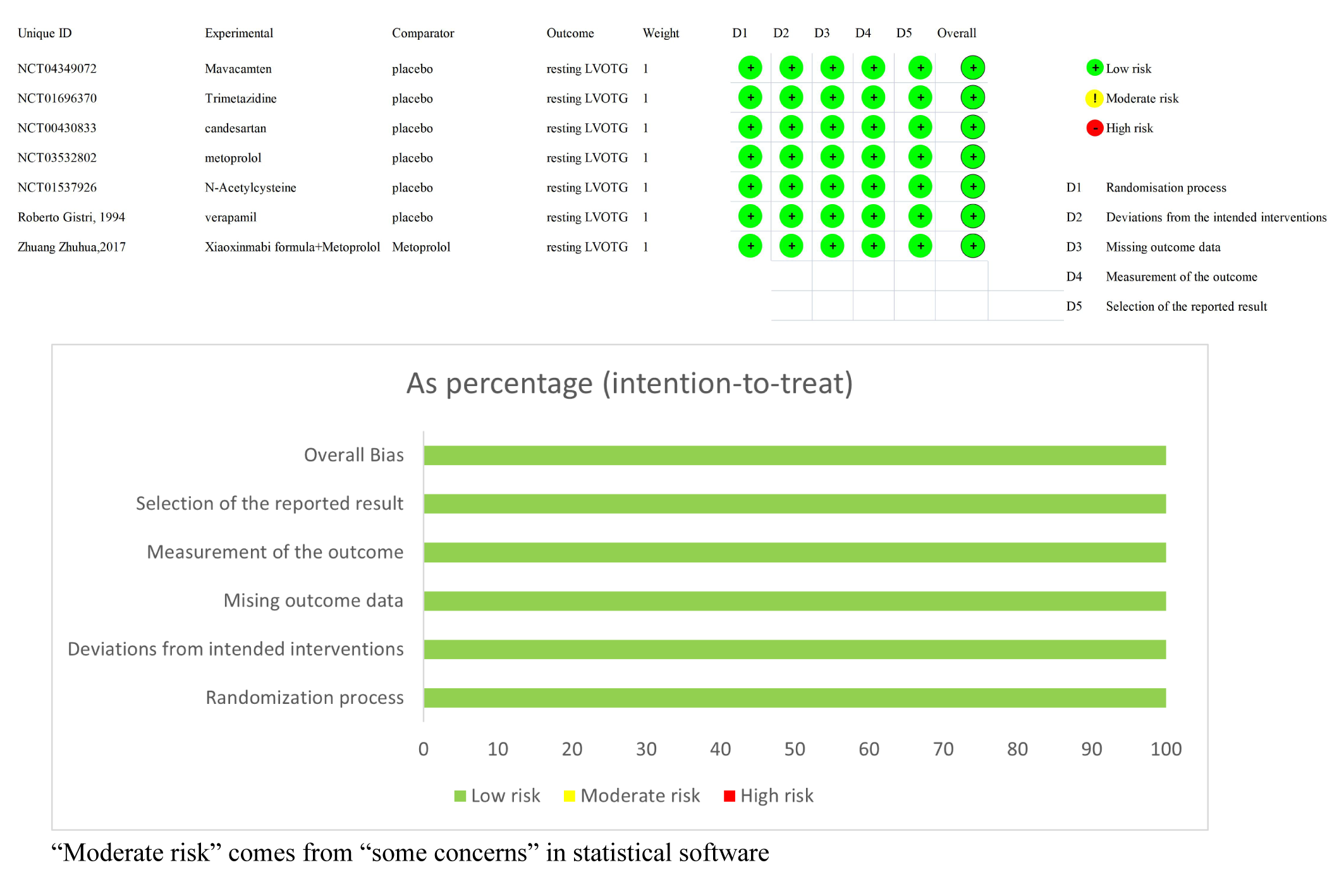

Supplement: Supplementary Figure S1 — Summary of bias risk. [file Image1.tif]
